# Impact of annual health check-ups on improvement in hypertension and abnormalities of glucose and lipid metabolism

**DOI:** 10.1038/s41440-025-02465-9

**Published:** 2025-11-21

**Authors:** Satoko Kameda, Hisaki Makimoto, Takeshi Fujiwara, Tomohiro Kikuchi, Takahide Kohro, Hiroshi Miyashita, Kazuomi Kario

**Affiliations:** 1 Data Science Centre, Jichi Medical University, Shimotsuke, Japan; 2 Department of Paediatrics, Shin-Oyama City Hospital, Oyama, Japan; 3 Cardiovascular Centre, Jichi Medical University, Shimotsuke, Japan; 4 The Institute of Medical Science, The University of Tokyo, Tokyo, Japan; 5 Departments of Internal Medicine and Health Development, Minamiuonuma City Hospital, Minamiuonuma, Japan

**Keywords:** Diabetes Mellitus, Dyslipidaemia, Health check-ups, Hypertension, Implementation hypertension

## Abstract

This study evaluated the 1-year improvement in blood pressure (BP), glucose metabolism (GM), and lipid metabolism (LM) abnormalities detected during health check-ups, and identified factors associated with improvement. This retrospective cohort study used data from a University Health Care Centre between April 2008 and March 2023. Adults with BP, GM, or LM abnormalities at baseline who attended the following year’s check-ups were included. The abnormalities were defined according to the criteria of the Japan Society of Ningen Dock. The primary outcome was improvement in abnormalities at the subsequent check-up, defined as no longer meeting the abnormality criteria. Multivariable logistic regression was used to examine factors associated with improvement. We analysed 2727 participants with BP abnormalities (mean age: 55.4 ± 9.0 years; 71.1% males), 1506 with GM (55.8 ± 8.5 years; 74.9% males), and 3793 with LM (52.4 ± 8.7 years; 61.0% males). Improvement occurred in 57.4% (BP), 29.3% (GM), and 57.5% (LM). Use of corresponding medication at baseline (odds ratios [ORs]: 0.38, 0.33, and 0.14, for BP, GM, and LM, respectively, *p* < 0.05) and 1-year weight loss (ORs: 1.08, 1.24, and 1.12, respectively, *p* < 0.05) were associated with improvement across all three domains, whereas use of corresponding medication at the subsequent check-up was associated with improvement in BP and LM (ORs: 2.22 and 14.97, respectively, *p* < 0.001). In this large health check-up cohort, over half of BP and LM, and approximately one-third of GM abnormalities improved within 1 year. Associations between improvement and both weight reduction and pharmacological treatment highlight the importance of lifestyle modification and timely medical management.

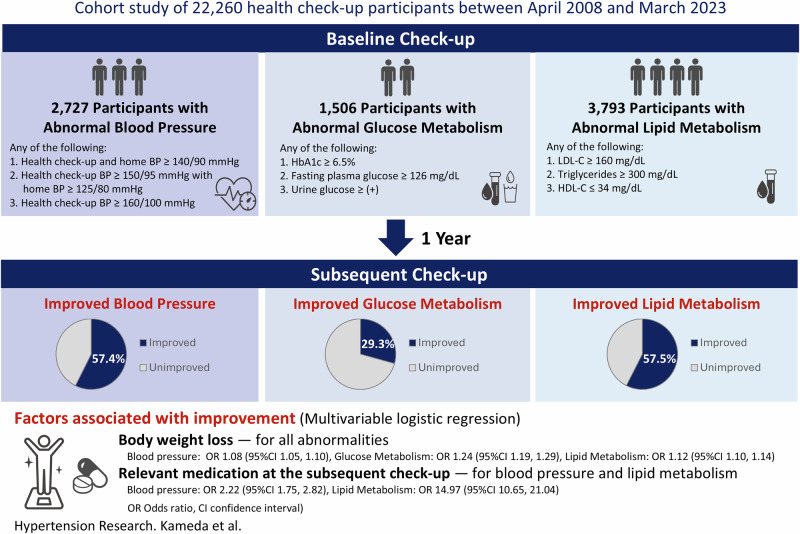

## Introduction

Periodic health checks are routine components of healthcare in many countries. In Japan, health check-up systems, including infant and toddler check-ups, school health examinations, workplace screenings, and specific health check-ups for adults, are widely implemented across the course of life [1]. These systems are well established in daily life, and ~70% of adults undergo annual check-up [2].

Among adults, health check-ups are intended to facilitate early detection and management of various diseases, particularly cardiovascular disease (CVD) risk factors. CVD is a leading cause of death worldwide [3], with hypertension, diabetes mellitus, and dyslipidaemia among the principal modifiable risks [4, 5]. Accordingly, comprehensive health check-ups routinely measure blood pressure and obtain laboratory indices of glucose and lipid metabolism, thereby providing opportunities to initiate lifestyle modification and medical treatment.

Nevertheless, robust evidence regarding the extent to which check-ups are followed by improved control of CVD risk factors in the general population is limited. Prior studies have often examined subsequent control among individuals newly identified and untreated for hypertension [6–8], impaired glucose metabolism [9], or dyslipidaemia [10]. Few investigations have included individuals already receiving treatment, assessed multiple risk domains within the same cohort in a real-world health check-up setting, or applied standardised definitions of improvement.

To address these gaps, we evaluated 1-year improvement in abnormalities of blood pressure, glucose metabolism, and lipid metabolism detected at annual health check-ups using the Japan Society of Ningen Dock criteria, and identified factors associated with this improvement, irrespective of treatment status.

## Methods

### Participants

This retrospective cohort study included adults aged ≥20 years who underwent health check-ups at Jichi Medical University Health Care Centre between April 1, 2008, and March 31, 2023. These were not mandatory occupational health check-ups, but rather voluntarily checks undertaken by individuals. When multiple check-up records were available for the same individual within the same fiscal year, only data from the first check-up were used. Records with missing data on blood pressure, glucose metabolism, or lipid metabolism were excluded.

The study population comprised individuals with blood pressure, glucose metabolism, or lipid metabolism abnormalities during a health check-up and who underwent a subsequent check-up in the following year. Abnormalities were assessed according to the criteria used at our healthcare centre, which were based on the classification criteria of the Japan Society of Ningen Dock and Preventive Medical Care [11]. In this study, findings that required further examination or treatment were considered abnormal. Specifically, blood pressure abnormality was defined any one of the following: (1) Both health check-up and home blood pressure of ≥140/90 mmHg; (2) Health check-up blood pressure of ≥150/95 mmHg with a home blood pressure of ≥125/80 mmHg or missing home blood pressure; or (3) Health check-up blood pressure of ≥160/100 mmHg. Glucose metabolism abnormality was defined any of the following: (1) haemoglobin A1c (HbA1c) of ≥6.5%, according to the National Glycohemoglobin Standardization Program; (2) fasting plasma glucose ≥126 mg/dL; or (3) urine glucose ≥(+). Lipid metabolism abnormality was defined any of the following: (1) low-density lipoprotein cholesterol (LDL-C) of ≥160 mg/dL; (2) triglycerides of ≥300 mg/dL; or (3) high-density lipoprotein cholesterol (HDL-C) of ≤34 mg/dL. Medical consultation was recommended for untreated individuals with these abnormalities.

### Outcomes

The primary outcome was the proportion of individuals whose abnormalities of blood pressure, glucose metabolism, or lipid metabolism improved at the subsequent check-up, which was defined as no longer meeting the criteria for abnormality described above. Factors associated with improvement at the subsequent health check-up were also examined.

### Variables

The following variables were collected: demographic characteristics, anthropometric measurements, responses to self-administered questionnaires on health-related behaviours and medical histories, systolic blood pressure (SBP) and diastolic blood pressure (DBP), and blood and urine test results. Body mass index (BMI) was calculated as weight in kilograms divided by the square of height in meters. Weight loss (%) was calculated as follows: (body weight at the baseline check-up − body weight at the subsequent check-up)/body weight at the baseline check-up × 100, with positive values indicating weight loss and negative values indicating weight gain. Lifestyle-related behaviours included current smoking status, exercise habits, and daily alcohol intake. Exercise habits were defined as engaging in moderate exercise for at least 30 min per session, at least twice a week, for >1 year. Daily alcohol intake was defined as daily consumption of alcohol, regardless of the amount. Medical histories included self-reported use of antihypertensive, antidiabetic, or antihyperlipidaemic medications. In addition, we assessed medical institution visits after the baseline check-up, as confirmed through response letters from consulting physicians, for individuals who were not receiving relevant medication at the baseline check-up.

### Statistical analysis

Analyses were conducted using Stata software version 19.0 (Stata Corp LP, College Station, TX, USA), and a *p*-value of <0.05 was considered statistically significant. Descriptive statistics were used to summarise baseline characteristics. Categorical variables are described as numbers with percentages, and continuous variables are described as means with standard deviation (SD). Changes in blood pressure and blood test results related to glucose and lipid metabolism between baseline and the subsequent check-up were assessed using paired *t*-tests. Multivariable logistic regression analysis was performed to examine factors associated with improvement at the subsequent health check-up. Explanatory variables included the following: baseline characteristics (age, sex, obesity status [BMI < 25 or ≥25 kg/m^2^], SBP, HbA1c, LDL-C, HDL-C, triglycerides, use of antihypertensive, antidiabetic, and/or antihyperlipidaemic medication), weight loss, and variables at the subsequent check-up (current smoking status, exercise habit, daily alcohol intake, and use of relevant medication). For each factor, odds ratio (OR) and 95% confidence interval (CI) were calculated. To evaluate potential multicollinearity among covariates, we calculated variance inflation factors (VIFs) using ordinary least squares regression, including all variables entered into multivariable logistic models.

For sensitivity analysis, we repeated the same analyses after excluding individuals who were receiving relevant medication at the baseline check-up.

### Ethics statement

The study protocol was approved by our institutional review board. In accordance with the Ethical Guidelines for Medical and Health Research Involving Human Subjects in Japan, informed consent was obtained using an opt-out approach, whereby information about the study was disclosed on the institution’s website, and participants were given the opportunity to decline participation.

## Results

### Participants and baseline characteristics

**Fig. 1:**
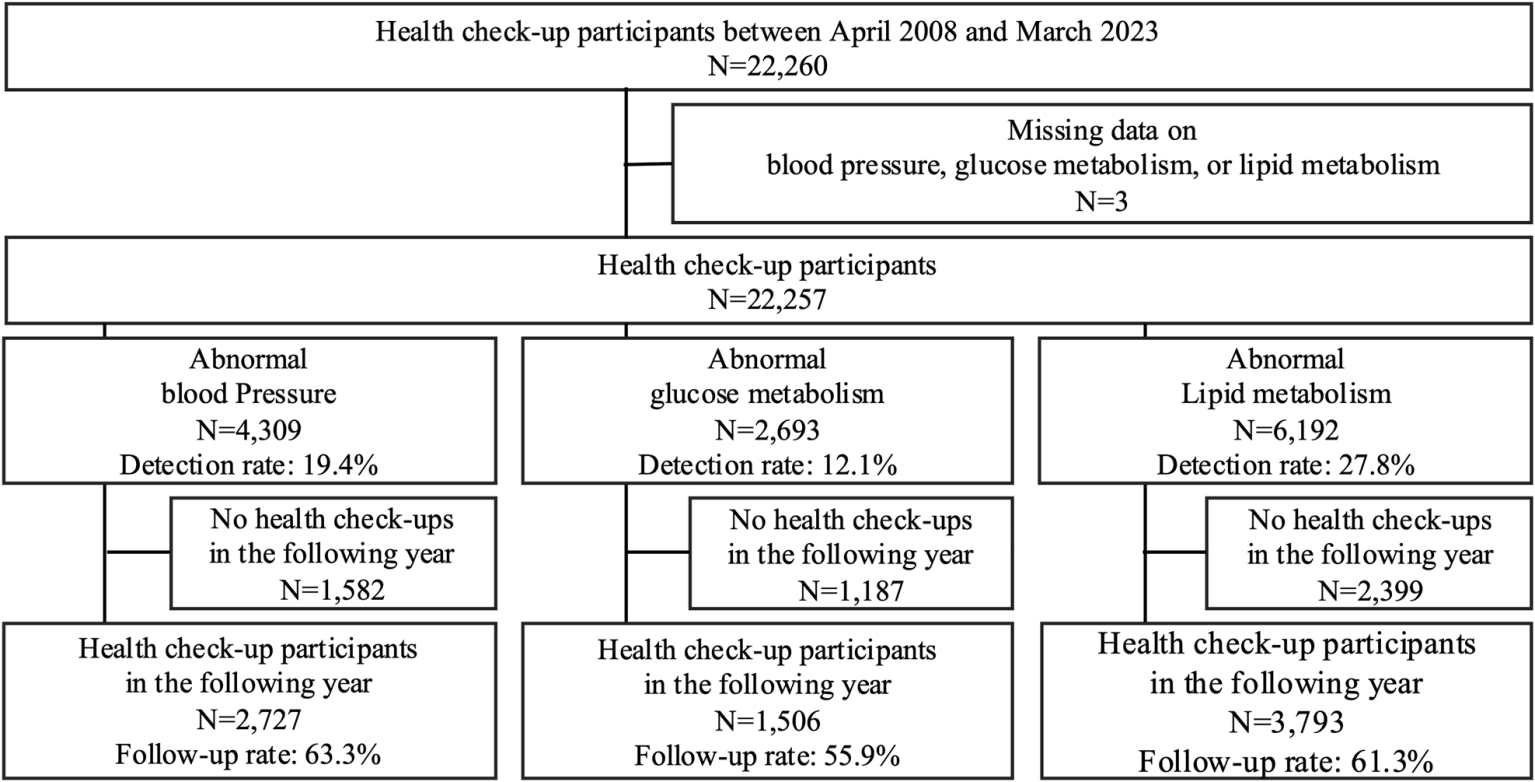
Flowchart defining the study cohort

Figure 1 shows a flowchart of the study cohort. A total of 22,260 individuals underwent health check-ups between April 2008 and March 2023. Of these, 2727 participants with blood pressure abnormalities, 1506 with glucose metabolism abnormalities, and 3793 with lipid metabolism abnormalities were included in the analysis after applying the eligibility criteria.

Table 1 shows the baseline characteristics of the participants. Participants with blood pressure abnormalities had a mean age of 55.4 ± 9.0 years, 71.1% were males, and 35.1% were on antihypertensive medication. Participants with glucose metabolism abnormalities had a mean age of 55.8 ± 8.5 years, 74.9% were males, and 25.6% were on antidiabetic medication. Participants with lipid metabolism abnormalities had a mean age of 52.4 ± 8.7 years, 61.0% were males, and 8.4% were on antihyperlipidaemic medication. The proportion of participants with obesity (BMI ≥ 25 kg/m^2^) was 46.5% for blood pressure abnormalities, 54.5% for glucose metabolism abnormalities, and 39.0% for lipid metabolism abnormalities, whereas the proportion of underweight individuals (BMI < 18.5 kg/m^2^) was 1.7%, 1.9%, and 1.7%, respectively.

**Table 1:** Baseline characteristics of participants

	Participants with abnormal BP	Participants with abnormal GM	Participants with abnormal LM
	*N* = 2727	*N* = 1506	*N* = 3793
	*n* (%) or mean (SD)	*n* (%) or mean (SD)	*n* (%) or mean (SD)
Age (years)	55.4 (9.0)	55.8 (8.5)	52.4 (8.7)
Male	1940 (71.1%)	1128 (74.9%)	2313 (61.0%)
Obesity (BMI ≥ 25 kg/m2)	1269 (46.5%)	821 (54.5%)	1480 (39.0%)
Waist circumference (cm)	88.9 (9.6)	91.3 (10.4)	87.1 (8.8)
SBP (mmHg)	153.1 (11.1)	133.7 (16.7)	127.7 (16.3)
DBP (mmHg)	95.8 (7.9)	83.5 (11.0)	80.2 (11.1)
HbA1c (%)	5.8 (0.8)	6.9 (1.1)	5.8 (0.8)
LDL-C (mg/dL)	128.7 (30.1)	127.5 (31.3)	161.6 (28.8)
HDL-C (mg/dL)	62.8 (17.0)	57.8 (14.9)	60.3 (16.8)
TG (mg/dL)	132.5 (87.3)	144.5 (93.8)	168.3 (125.8)
Smoking			
Never smoked	1263 (46.3%)	565 (37.5%)	1938 (51.1%)
Current smoking	441 (16.2%)	382 (25.4%)	782 (20.6%)
Quit smoking	1011 (37.1%)	551 (36.6%)	1066 (28.1%)
Missing	12 (0.4%)	8 (0.5%)	7 (0.2%)
Antihypertensive medication use			
Yes	956 (35.1%)	611 (40.6%)	631 (16.6%)
No	1737 (63.7%)	868 (57.6%)	3111 (82.0%)
Missing	34 (1.2%)	27 (1.8%)	51 (1.3%)
Antidiabetic medication use			
Yes	189 (6.9%)	385 (25.6%)	150 (4.0%)
No	2446 (89.7%)	1080 (71.7%)	3564 (94.0%)
Missing	92 (3.4%)	41 (2.7%)	79 (2.1%)
Antihyperlipidemic medication use			
Yes	496 (18.2%)	420 (27.9%)	317 (8.4%)
No	2140 (78.5%)	1026 (68.1%)	3398 (89.6%)
Missing	91 (3.3%)	60 (4.0%)	78 (2.1%)

*BMI* body mass index, *BP* blood pressure, *DBP* diastolic blood pressure, *FBS* fasting blood sugar, *GM* glucose metabolism, *HbA1c* haemoglobin A1c, *HDL-C* high-density lipoprotein cholesterol, *LDL-C* low-density lipoprotein cholesterol, *LM* lipid metabolism, *SD* standard deviation, *SBP* systolic blood pressure, *TG* triglycerides

### Blood pressure abnormalities

**Fig. 2:**
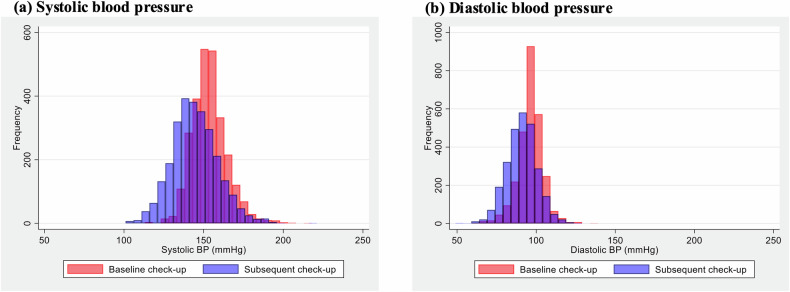
Distribution of blood pressure at baseline and the subsequent check-up among individuals with blood pressure abnormalities. Red bars represent baseline measurements and blue bars represent subsequent check-up measurements of **a** systolic and **b** diastolic blood pressures. BP blood pressure

**Fig. 3:**
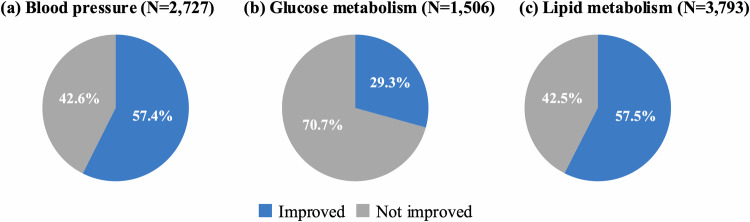
Proportion of individuals with improvement in abnormalities at the subsequent check-up. Blue segments represent participants who showed improvements in abnormalities, and grey segments represent those who did not show improvement in abnormalities of **a** blood pressure, **b** glucose metabolism, and **c** lipid metabolism

**Fig. 4:**
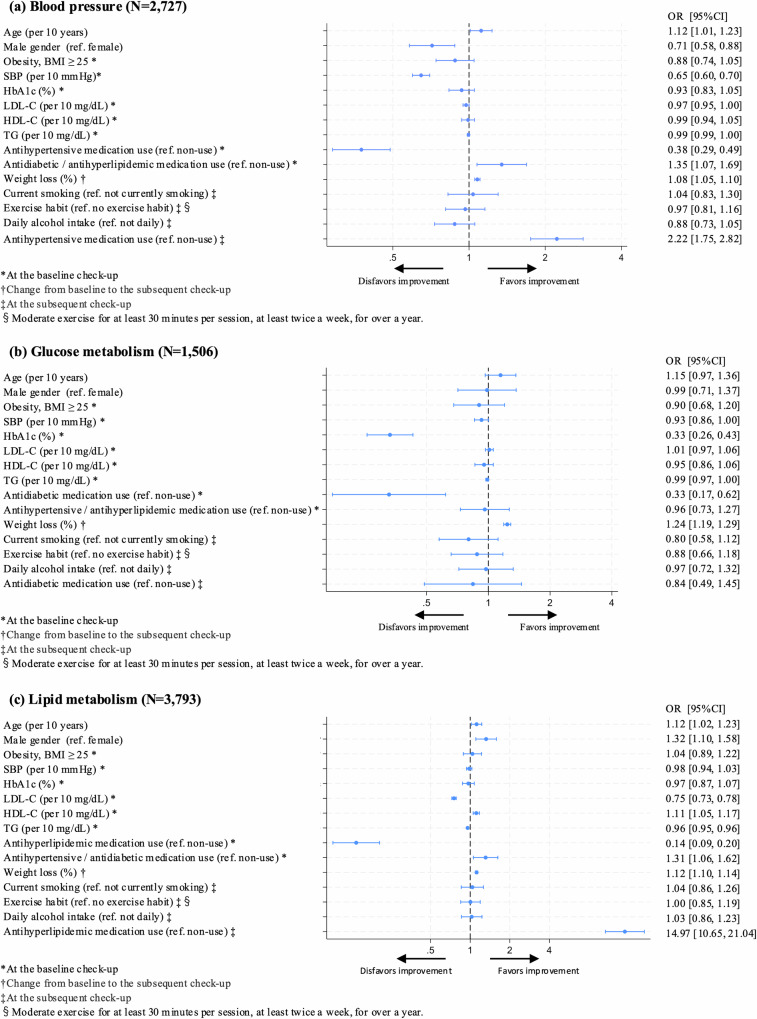
Factors associated with improvement in abnormalities at the subsequent check-up. Odds ratios for improvement in abnormalities at the subsequent check-up were calculated using logistic regression models for **a** blood pressure, **b** glucose metabolism, and **c** lipid metabolism. The following variables were included as explanatory variables: baseline characteristics (age, sex, obesity status [BMI < 25 or ≥ 25 kg/m^2^], SBP, HbA1c, LDL-C, HDL-C, triglycerides, use of antihypertensive, antidiabetic and/or antihyperlipidaemic medications, weight loss, and variables at the subsequent check-up (current smoking status, exercise habits, daily alcohol intake, and use of relevant medication). *Variables at the baseline check-up. †Change from baseline to the subsequent check-up. ‡Variables at the subsequent check-up. §Exercise habit was defined as engaging in moderate exercise for at least 30 min per session, at least twice a week, for >1 year. BMI body mass index, CI confidence interval, HbA1c haemoglobin A1c, HDL-C high-density lipoprotein cholesterol, LDL-C low-density lipoprotein cholesterol, OR odds ratio, SBP systolic blood pressure, TG triglycerides

Figure 2 shows blood pressure distribution at baseline and at the subsequent check-up among the 2727 individuals with blood pressure abnormalities. The mean SBP was 153.1 ± 11.1 mmHg and DBP was 95.8 ± 7.9 mmHg. Both the SBP and DBP decreased significantly by 8.6 mmHg (95% CI, 8.0–9.1; *p* < 0.001) and 5.1 mmHg (95% CI, 4.7–5.4; *p* < 0.001), respectively, at the subsequent check-up. Among individuals with baseline SBP of ≥160 mmHg, the reduction was more pronounced, with SBP decreasing by 17.2 mmHg (95% CI, 16.0–18.4; *p* < 0.001; Supplementary Fig. 1). Overall, 57.4% of the individuals showed improvement in blood pressure abnormalities at the subsequent check-up (Fig. 3). Figure 4 shows the factors associated with improvement at the subsequent check-up, based on a logistic regression analysis. Improvement in blood pressure abnormalities was significantly associated with older age (OR, 1.12 [per 10 years]; 95% CI, 1.01–1.23; *p* = 0.03), female sex (OR, 0.71 for male sex; 95% CI, 0.58–0.88; *p* = 0.001), lower baseline SBP (OR, 0.65 [per 10 mmHg]; 95% CI, 0.60–0.70; *p* < 0.001), non-use of antihypertensive medication at the baseline check-up (OR, 0.38 for use of antihypertensive medication at the baseline check-up; 95% CI, 0.29–0.49; *p* < 0.001), use of antidiabetic and/or antihyperlipidaemic medication at the baseline check-up (OR, 1.35; 95% CI, 1.07–1.69; *p* = 0.010), weight loss (OR, 1.08; 95% CI, 1.05–1.10; *p* < 0.001), and use of antihypertensive medication at the subsequent check-up (OR, 2.22; 95% CI, 1.75–2.82; *p* < 0.001). Mean (range) VIF for this model was 1.35 (1.02–2.24), indicating that multicollinearity was unlikely to have affected the results.

### Glucose metabolism abnormalities

Among the 1506 individuals with glucose metabolism abnormalities, the distribution of HbA1c at baseline and the subsequent check-up largely overlapped (Supplementary Fig. 2), and there was no significant difference observed (mean difference, 0.02%; 95% CI, −0.02 to 0.07, *p* = 0.336). However, among 385 individuals with HbA1c of ≥7.0% at the baseline check-up, HbA1c decreased significantly by 0.3% (95% CI, 0.17–0.47; *p* < 0.001). Overall, 29.3% of the individuals showed improvement in glucose metabolism abnormalities at the subsequent health check-up (Fig. 3). Logistic regression analysis showed that lower baseline HbA1c (OR, 0.33 for HbA1c; 95% CI, 0.26–0.43; *p* < 0.001), non-use of antidiabetic medication at the baseline check-up (OR, 0.33 for use of antidiabetic medication at the baseline check-up; 95% CI, 0.17–0.62; *p* = 0.001), and weight loss (OR, 1.24; 95% CI, 1.19–1.29; *p* < 0.001) were significantly associated with improvement in glucose metabolism abnormalities at the subsequent check-up (Fig. 4). Mean (range) VIF for this model was 1.45 (1.06–3.03), indicating that multicollinearity was unlikely to have affected the results.

### Lipid metabolism abnormalities

Among the 3793 individuals with lipid metabolism abnormalities, 3135 (82.7%) had LDL-C abnormalities, 572 (15.1%) had triglyceride abnormalities, 181 (4.8%) had HDL-C abnormalities, and 95 had multiple lipid abnormalities. LDL-C and triglycerides levels decreased significantly from baseline to the subsequent check-up by 18.5 mg/dL (95% CI, 17.5–19.5; *p* < 0.001) and 154.9 mg/dL (95% CI, 138.7–171.1; *p* < 0.001), respectively, while HDL-C significantly increased by 4.5 mg/dL (95% CI, 3.7–5.3; *p* < 0.001) (Supplementary Fig. 3). At the subsequent check-up, 57.5% of the participants showed improvement in lipid metabolism abnormalities (Fig. 3), including 57.7% of those with LDL-C abnormalities, 73.1% with triglyceride abnormalities, and 67.4% with HDL-C abnormalities. Logistic regression analysis showed that older age (OR, 1.12 [per 10 years]; 95% CI, 1.02–1.23; *p* = 0.016), male sex (OR, 1.32; 95% CI, 1.10–1.58; *p* = 0.002), lower baseline LDL-C (OR, 0.75 [per 10 mg/dL]; 95% CI, 0.73–0.78; *p* < 0.001) or triglyceride (OR, 0.96 [per 10 mg/dL]; 95% CI, 0.95–0.96; *p* < 0.001), higher baseline HDL-C (OR, 1.11 [per 10 mg/dL]; 95% CI, 1.05–1.17; *p* < 0.001), non-use of antihyperlipidaemic medication at the baseline check-up (OR, 0.14 for use of antihyperlipidaemic medication at the baseline check-up; 95% CI, 0.09–0.20; *p* < 0.001), use of antihypertensive and/or antidiabetic medication at the baseline check-up (OR, 1.31; 95% CI, 1.06–1.62; *p* = 0.013), weight loss (OR, 1.12; 95% CI, 1.10–1.14; *p* < 0.001), and use of antihyperlipidaemic medication at the subsequent check-up (OR, 14.97; 95% CI, 10.65–21.04; *p* < 0.001) were significantly associated with improvement in lipid metabolism abnormalities at the subsequent check-up (Fig. 4). When LDL-C and triglyceride abnormalities were analysed separately, the use of antihyperlipidaemic medication at both baseline and subsequent check-up was significantly associated with improvement in LDL-C (baseline: OR, 0.10; 95% CI, 0.06–0.17; *p* < 0.001; subsequent check-up: OR, 30.34; 95% CI, 19.49–47.25; *p* < 0.001), whereas no significant association was observed for triglyceride abnormalities (baseline: OR, 0.57; 95% CI, 0.26–1.27; *p* = 0.169; subsequent check-up: OR, 1.39; 95% CI, 0.69–2.82; *p* = 0.357). However, weight loss was significantly associated with improvement in both LDL-C (OR, 1.1; 95% CI, 1.09–1.14; *p* < 0.001) and triglyceride (OR, 1.16; 95% CI, 1.09–1.24; *p* < 0.001, Supplementary Fig. 4). Mean (range) VIF for this model was 1.35 (1.02–1.93), indicating that multicollinearity was unlikely to have affected the results.

### Sensitivity analysis

A total of the 1771 participants with blood pressure abnormalities, 1121 with glucose metabolism abnormalities, and 3476 with lipid metabolism abnormalities were included in the sensitivity analysis, excluding individuals who were receiving relevant medications at the baseline check-up. Among these participants, 36.8% visited a medical institution after the baseline check-up for abnormal blood pressure, 44.3% for abnormal glucose metabolism, and 34.8% for abnormal lipid metabolism. The baseline characteristics are shown in Supplementary Table 1. The proportions of individuals whose abnormalities improved at the subsequent check-up were 58.3% for blood pressure, 36.8% for glucose metabolism, and 56.7% for lipid metabolism (Supplementary Fig. 5). Weight loss was consistently associated with improvement in blood pressure (OR, 1.09; 95% CI, 1.06–1.12), glucose metabolism (OR, 1.25; 95% CI, 1.20–1.30), and lipid metabolism (OR, 1.12; 95% CI, 1.10–1.15) at the subsequent check-up (all *p* < 0.001), and the use of corresponding medication at the subsequent check-up was also significantly associated with improvement in blood pressure (OR, 2.38; 95% CI, 1.84–3.08; *p* < 0.001) and lipid metabolism (OR, 22.73; 95% CI, 15.11–34.21; *p* < 0.001), respectively (Supplementary Fig. 6).

## Discussion

This observational study evaluated 1-year changes after abnormalities in blood pressure, glucose metabolism, and lipid metabolism were identified during routine health check-ups. At the next annual check-up, the improvements were observed in 57.4% of individuals with blood pressure abnormalities, 29.3% of those with glucose metabolism, and 57.5% of those with lipid metabolism. Weight loss and non-use of relevant medications at baseline were consistently associated with improvement across all three domains, and the reported use of relevant medications at the subsequent check-up was also associated with improvement in blood pressure and lipid metabolism.

Previous studies examining follow-up control of blood pressure, glucose, and lipid metabolism abnormalities have yielded heterogeneous estimates owing to differences in populations and methods [6–10]. Among participants untreated for hypertension at baseline, one study reported improvement in 58.4% of 5428 individuals at follow-up [8], and another reported improvement in 52% among those on antihypertensive medication at follow-up versus 37% among those not on medication [7]. For lipid metabolism, improvement was 46% among those who started antihyperlipidaemic therapy compared with 23% among those who did not [10]. Regarding glucose metabolism, a small study among untreated individuals reported persistent abnormalities in 72% (36/50) of men and 40% (4/10) of women [9]. In our mixed population of treated and untreated participants in voluntary health check-ups, the observed improvement proportions were comparable with or slightly higher than those in previous reports on blood pressure and lipid metabolism. However, in both prior work and our study, improvement was achieved in only approximately half or few participants with blood pressure and lipid metabolism abnormalities, and in less than one-third of those with glucose metabolism abnormality, underscoring the need for structured follow-up strategies.

Non-use of the relevant medication at baseline and weight loss were consistently associated with improvement across all abnormalities. A previous study showed that newly identified hypertension (no history of hypertension at the preceding check-up) was associated with achievement of target blood pressure [8]. Together, these findings suggest that previously undiagnosed or untreated abnormalities may be easier to improve through lifestyle changes or treatment initiation, whereas abnormalities under treatment may be more difficult to improve. Previous studies have also shown that obesity is associated with poorer subsequent control of blood pressure and lipid metabolism abnormalities [7, 10], and that weight loss in individuals with obesity improves blood pressure, glucose metabolism, and lipid metabolism abnormalities [12–14]. In our analysis stratified by obesity status, weight loss was significantly associated with an improvement in all three abnormalities in both participants with and without obesity (data not shown), underscoring the importance of weight management, regardless of baseline obesity status.

For blood pressure and lipid metabolism abnormalities, the use of relevant medications at the subsequent check-up showed the strongest association with improvement in our logistic regression analysis. This association was even stronger in the sensitivity analysis, which was restricted to participants who were untreated at baseline. Consistent with our findings, previous studies have reported higher improvement rates among those who initiated medication after health check-ups than those among individuals who did not: for blood pressure, 52.1% vs. 37.1% in one study [7] and 61.6% vs. 55.9% in another study [8]; and for lipid metabolism, 45.6% vs. 22.7% [10], respectively. These results suggest the importance of ensuring that those with abnormalities detected during health check-ups receive appropriate follow-up care and treatment. Furthermore, in an additional analysis separating LDL-C and triglyceride abnormalities, the use of antihyperlipidaemic medication at the subsequent check-up was significantly associated with an improvement in LDL-C abnormalities, whereas no such association was observed for triglyceride abnormalities. These findings suggest that LDL-C management can be effectively achieved with pharmacological therapy, whereas triglyceride abnormalities are more difficult to improve, reflecting the multifactorial and complex nature of triglyceride metabolism [15, 16].

In addition, ongoing treatment for other conditions may facilitate timely action in response to new findings. Participants who had already started medications for glucose or lipid metabolism abnormalities had a higher proportion of improved blood pressure. Similarly, participants who had already started medications for blood pressure or glucose metabolism abnormalities had a higher proportion of improved lipid metabolism.

Applying a common analytical approach across the three domains highlighted the differences in their trajectories. The proportion of improvement was lowest for glucose metabolism. In contrast, the proportion of individuals who sought follow-up medical care was highest in this group in the sensitivity analysis. Notably, the use of antidiabetic medication at the subsequent check-up was not significantly associated with improvement in glucose metabolism, suggesting that glucose metabolism abnormalities may be more challenging to improve through medical intervention than blood pressure or lipid metabolism. For blood pressure and LDL-C, aggressive lowering is generally recommended under the “lower-the-better” principle [17, 18], whereas intensive glucose lowering can increase the risk of severe hypoglycaemia and may be harmful [19]. Therefore, medical interventions for glucose metabolism are managed more conservatively, which may partly explain the lower proportion of improvements in these abnormalities.

This study has several limitations. First, the participants were voluntary health check-up attendees who may have been more health-conscious than the general population, introducing selection bias and limiting generalisability. Second, detailed information on medical interventions, including the number, class, dose, and duration of prescribed drugs, which could have affected the outcomes, was unavailable. In addition, unmeasured confounding factors, such as socioeconomic status, may have affected the results. Third, a 1-year interval may be insufficient to capture changes in glycaemic control. Fourth, our study population included a high proportion of individuals with obesity and few individuals with underweight; therefore, the results should be interpreted with caution when applied to underweight populations. Nevertheless, the high prevalence of obesity in our cohort reflects the real-world distribution of individuals with these CVD risk factors and is clinically relevant.

In conclusion, more than half of the participants with blood pressure or lipid metabolism abnormalities showed improvement at the next annual check-up, whereas approximately one-third of those with glucose metabolism abnormalities showed improvement. Importantly, weight loss was associated with improvement in all the three abnormalities. Regarding blood pressure and lipid metabolism abnormalities, medication at the subsequent check-up showed the strongest association with improvement. These findings support follow-up strategies that combine promotion of lifestyle changes, particularly weight reduction, with timely initiation of indicated medical interventions to enhance the effectiveness of health check-ups.

## Supplementary information

Supplementary information
