# Supplementary material for: Impact of annual health check-ups on improvement in hypertension and abnormalities of glucose and lipid metabolism
Source: Hypertens Res. 2025 Nov 21;49(2):516–25. doi: 10.1038/s41440-025-02465-9 (PMC12823388; doi:10.1038/s41440-025-02465-9)
Supplement: Supplementary file 1 — Supplementary information [file 41440_2025_2465_MOESM1_ESM.docx]

**Supplementary information**

Impact of Annual Health Checkups on Improvement in Hypertension and Abnormalities of Glucose and Lipid Metabolism

Satoko Kameda ^a,b*^, Hisaki Makimoto ^a,c,d*^, Takeshi Fujiwara ^c^, Tomohiro Kikuchi ^a^, Takahide Kohro ^a,c^, Hiroshi Miyashita ^a,e^, Kazuomi Kario ^c^

^a^Data Science Centre, Jichi Medical University, Shimotsuke, Japan

^b^Department of Paediatrics, Shin-Oyama City Hospital, Oyama, Japan.

^c^Cardiovascular Centre, Jichi Medical University, Shimotsuke, Japan

^d^Institute of Medical Science, University of Tokyo, Tokyo, Japan

^e^Department of Internal Medicine and Health Development, Minamiuonuma City Hospital, Minamiuonuma, Japan

***Table of Contents***

Supplementary Table 1. Baseline characteristics of untreated participants

Supplementary Figure 1. Distribution of blood pressure at baseline and the subsequent check-up among individuals with baseline systolic blood pressure of ≥160 mmHg (N=643)

Supplementary Figure 2. Distribution of HbA1c levels at baseline and the subsequent check-up among individuals with abnormalities of glucose metabolism

Supplementary Figure 3. Distribution of lipid parameters at baseline and the subsequent checkup among individuals with corresponding abnormalities

Supplementary Figure 4. Factors associated with improvements in LDL-C and triglyceride abnormalities at the subsequent check-up

Supplementary Figure 5. Proportion of individuals with improvement in abnormalities at the subsequent check-up among untreated participants at the baseline check-up

Supplementary Figure 6. Factors associated with improvement in abnormalities at the subsequent checkup in participants untreated at the baseline checkup

**Supplementary Table 1. Baseline characteristics of untreated participants**

|  | Participants with abnormal BP | Participants with abnormal GM | Participants with abnormal LM |
| --- | --- | --- | --- |
|  | N=1,771 | N=1,121 | N=3,476 |
|  | n (%) or mean (SD) | n (%) or mean (SD) | n (%) or mean (SD) |
| Age (years) | 53.8 (8.9) | 55.7 (8.5) | 52.0 (8.6) |
| Male | 1,245 (70.3%) | 831 (74.1%) | 2,083 (59.9%) |
| Obesity (BMI **≥**25 kg/m^2^) | 799 (45.1%) | 622 (55.5%) | 1,320 (38.0%) |
| Waist circumference (cm) | 88.5 (9.7) | 91.5 (10.5) | 86.9 (8.8) |
| SBP (mmHg) | 154.4 (11.2) | 134.1 (16.9) | 127.4 (16.4) |
| DBP (mmHg) | 97.1 (7.6) | 84.0 (11.2) | 80.1 (11.2) |
| HbA1c (%) | 5.7 (0.7) | 6.7 (1.1) | 5.7 (0.7) |
| LDL-C (mg/dL) | 132.1 (30.9) | 130.8 (30.9) | 162.7 (27.7) |
| HDL-C (mg/dL) | 63.1 (17.0) | 57.4 (14.6) | 60.6 (16.8) |
| TG (mg/dL) | 132.6 (91.4) | 150.0 (98.5) | 164.5 (124.5) |
| Smoking |  |  |  |
| Never smoked | 841 (47.5%) | 421 (37.6%) | 1,817 (52.3%) |
| Current smoking | 323 (18.2%) | 287 (25.6%) | 704 (20.3%) |
| Quit smoking | 602 (34.0%) | 405 (36.1%) | 949 (27.3%) |
| Missing | 5 (0.3%) | 8 (0.7%) | 6 (0.2%) |
| Antihypertensive medication use |  |  |  |
| Yes | NA | 417 (37.2%) | 486 (14.0%) |
| No | 1,737 (63.7%) | 690 (61.6%) | 2,948 (84.8%) |
| Missing | 34 (1.2%) | 14 (1.2%) | 42 (1.2%) |
| Antidiabetic medication use |  |  |  |
| Yes | 63 (3.6%) | NA | 98 (2.8%) |
| No | 1,677 (94.7%) | 1,080 (71.7%) | 3,313 (95.3%) |
| Missing | 31 (1.8%) | 41 (2.7%) | 65 (1.9%) |
| Antihyperlipidemic medication use |  |  |  |
| Yes | 173 (9.8%) | 257 (22.9%) | NA |
| No | 1,565 (88.4%) | 834 (74.4%) | 3,398 (97.8%) |
| Missing | 33 (1.9%) | 30 (2.7%) | 78 (2.2%) |
| *BMI,* body mass index; *BP,* blood pressure; *DBP,* diastolic blood pressure; *FBS,* fasting blood sugar; *GM,* glucose metabolism; *HbA1c,* hemoglobin A1c; *HDL-C,* high-density lipoprotein cholesterol; *LDL-C,* low-density lipoprotein cholesterol; *LM,* lipid metabolism; *NA,* not applicable; *SD,* standard deviation; *SBP,* systolic blood pressure; *TG,* triglycerides | | | |

**Supplementary Figure 1. Distribution of blood pressure at baseline and the subsequent check-up among individuals with baseline systolic blood pressure of ≥160 mmHg (N=643)**

Red bars represent baseline measurements, and blue bars represent subsequent check-up measurements for systolic blood pressure.

*BP,* blood pressure

**(a) All individuals with GM abnormalities (N=1,506)**

**(b) Individuals with HbA1c ≥ 7.0% at baseline (N=385)**

**Supplementary Figure 2. Distribution of HbA1c levels at baseline and the subsequent check-up among individuals with abnormalities of glucose metabolism**

Red bars represent baseline measurements, and blue bars represent subsequent check-up measurements for (a) all individuals with abnormalities of glucose metabolism and (b) those with HbA1c of ≥7.0% at baseline.

*GM,* glucose metabolism; *HbA1c,* hemoglobin A1c

**Supplementary Figure 3. Distribution of lipid parameters at baseline and the subsequent checkup among individuals with corresponding abnormalities**

**(a) LDL-C (N=3,135)**

**(b) Triglycerides (N=572)**

**(c) HDL-C (N=181)**

Red bars represent baseline measurements, and blue bars represent subsequent check-up measurements for individuals with abnormalities of (a) LDL-C, (b) triglyceride, and (c) HDL-C.

*LDL-C,* low-density lipoprotein cholesterol; *HDL-C,* high-density lipoprotein cholesterol


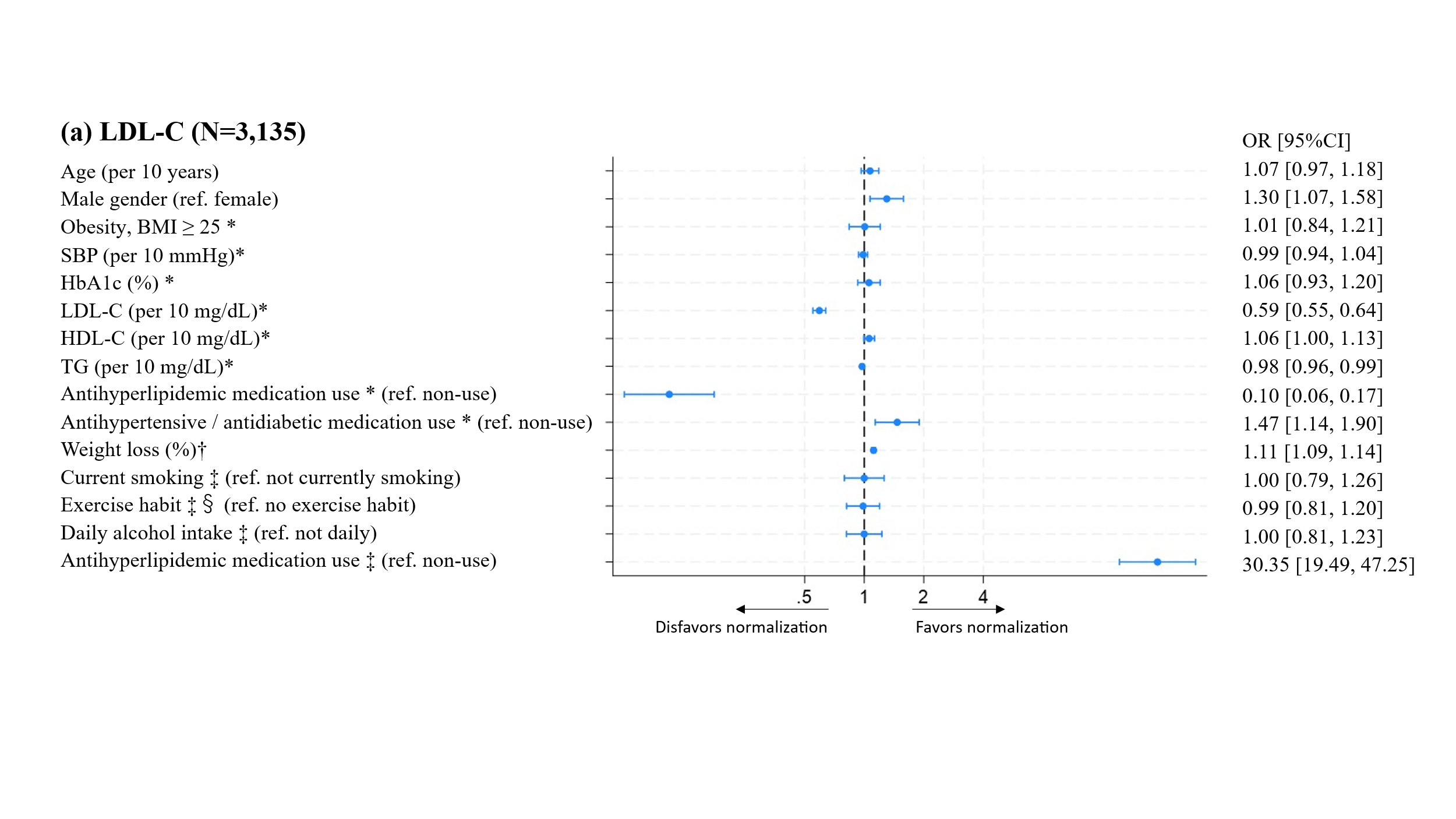


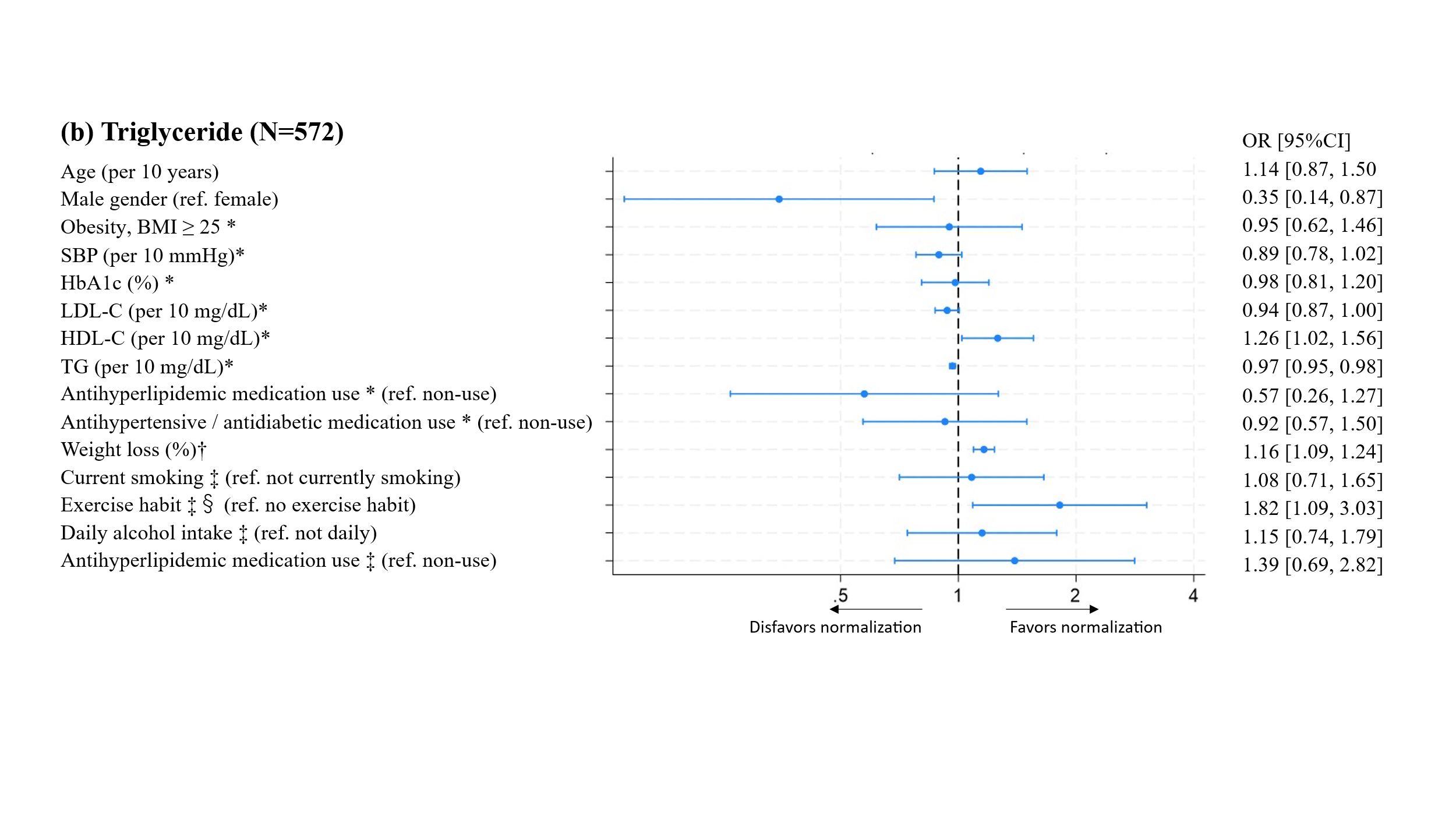


**Supplementary Figure 4. Factors associated with improvement in LDL-C and triglyceride abnormalities at the subsequent check-up**

Odds ratios for improvement in abnormalities at the subsequent check-up were calculated using logistic regression models for (a) LDL-C and (b) triglyceride. The following variables were included as explanatory variables: baseline characteristics (age, sex, obesity status [BMI <25 or ≥ 25 kg/m^2^], SBP, HbA1c, LDL-C, HDL-C, triglycerides, use of antihypertensive, antidiabetic, and/or antihyperlipidaemic medications), weight loss, and variables at the subsequent check-up (current smoking status, exercise habits, daily alcohol intake, and use of relevant medication).

*Variables at the baseline check-up. †Change from baseline to the subsequent check-up. ‡Variables at the subsequent check-up. §Exercise habit was defined as engaging in moderate exercise for at least 30 min per session, at least twice a week, for >1 year.

*BMI,* body mass index; *CI,* confidence interval; *HbA1c,* haemoglobin A1c; *HDL-C,* high-density lipoprotein cholesterol; *LDL-C,* low-density lipoprotein cholesterol; *OR,* odds ratio; *SBP,* systolic blood pressure; *TG,* triglycerides


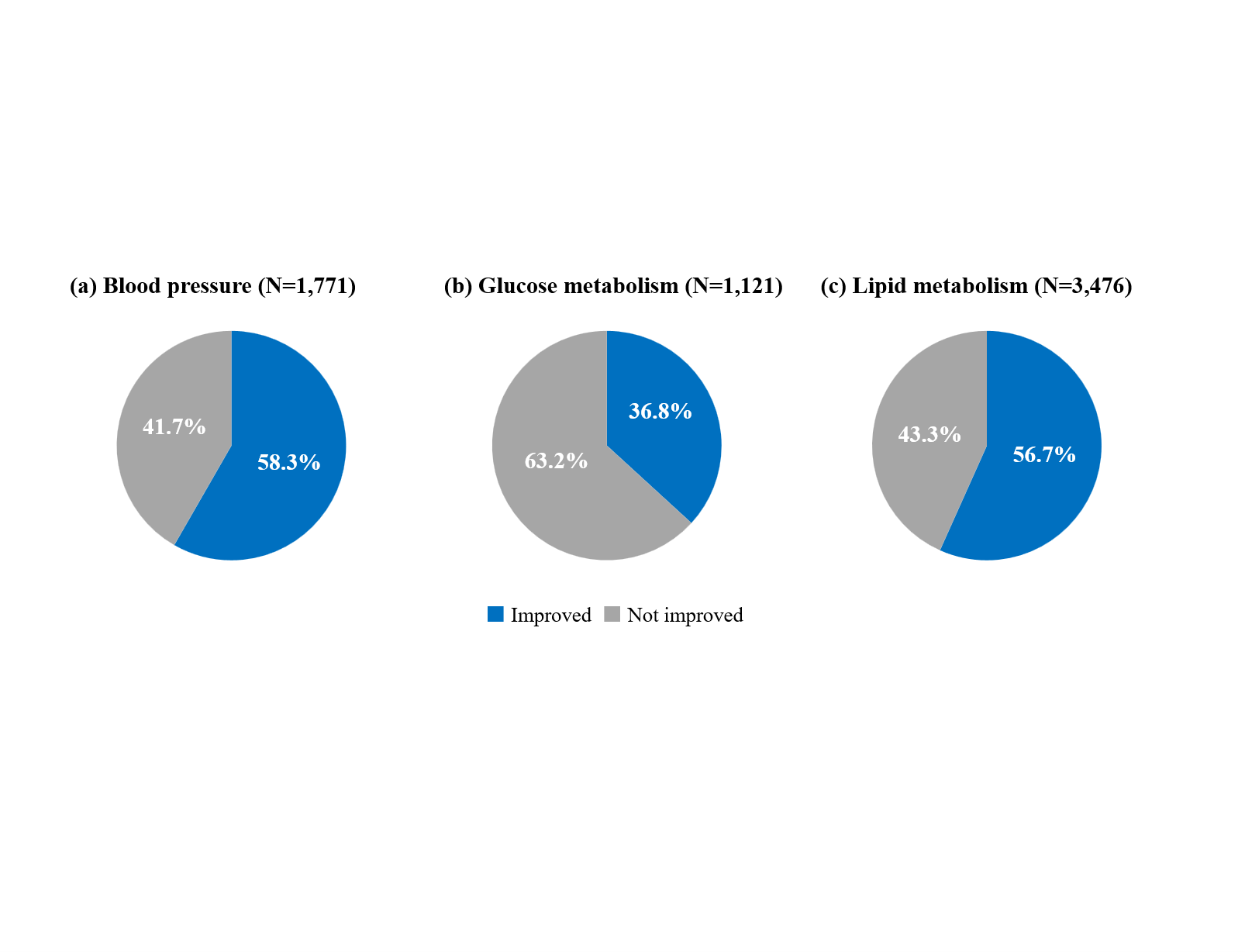


**Supplementary Figure 5. Proportion of individuals with improvement in abnormalities at the subsequent check-up among untreated participants at the baseline check-up**

Blue segments represent participants who showed improvements in abnormalities, and gray segments represent those who did not for (a) blood pressure, (b) glucose metabolism, and (c) lipid metabolism.


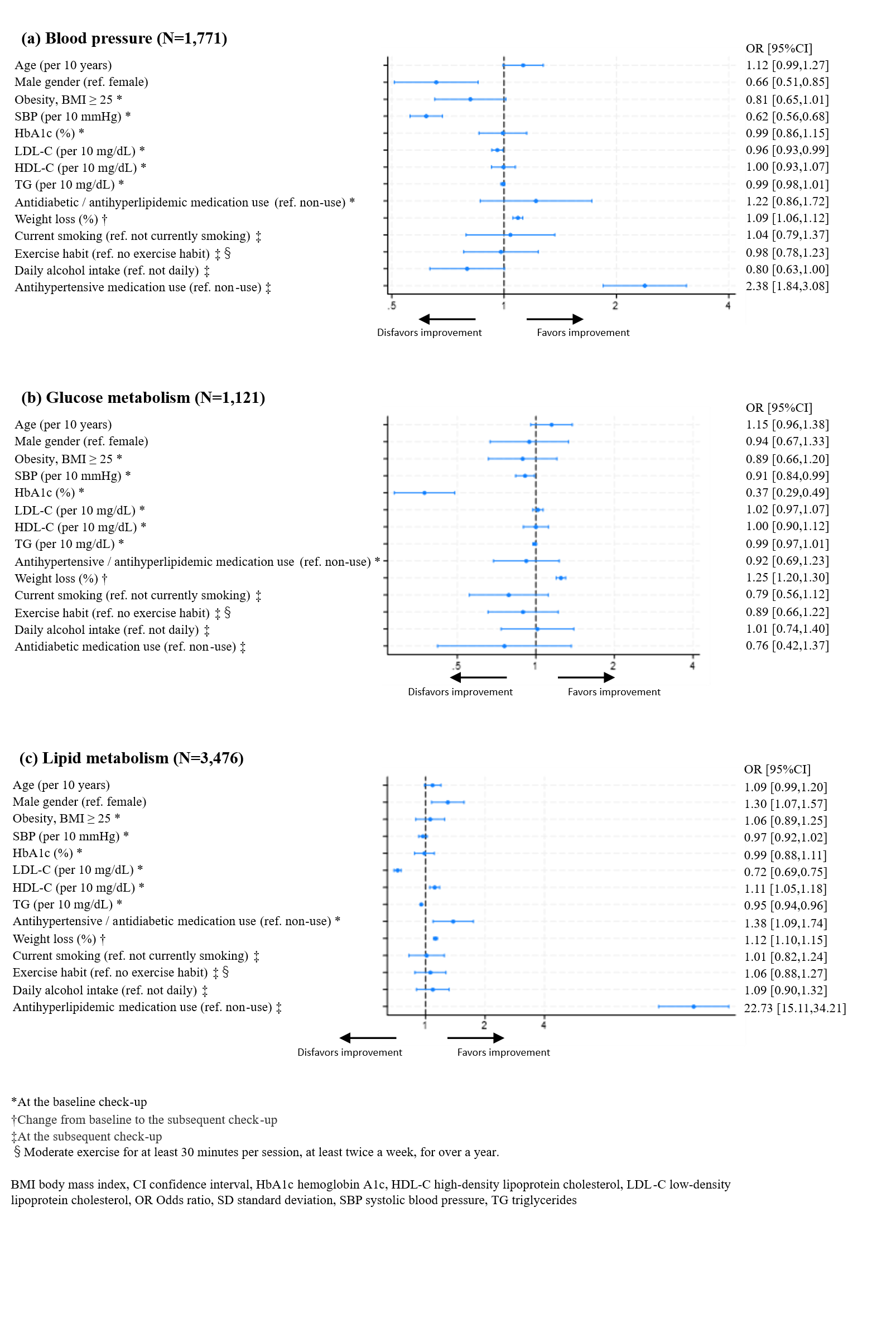


**Supplementary Figure 6. Factors associated with improvement in abnormalities at the subsequent checkup in participants untreated at the baseline checkup**

Odds ratios for improvement in abnormalities at the subsequent check-up were calculated using logistic regression models for (a) blood pressure, (b) glucose metabolism, and (c) lipid metabolism. The following variables were included as explanatory variables: baseline characteristics (age, sex, obesity status [BMI <25 or ≥25 kg/m^2^], SBP, HbA1c, LDL-C, HDL-C, triglycerides, use of antihypertensive, antidiabetic, and/or antihyperlipidaemic medications), weight loss, and variables at the subsequent check-up (current smoking status, exercise habits, daily alcohol intake, and use of relevant medication).

*Variables at the baseline check-up. †Change from baseline to the subsequent check-up. ‡Variables at the subsequent check-up. §Exercise habit was defined as engaging in moderate exercise for at least 30 min per session, at least twice a week, for >1 year.

*BMI,* body mass index; *CI,* confidence interval; *HbA1c,* haemoglobin A1c; *HDL-C,* high-density lipoprotein cholesterol; *LDL-C,* low-density lipoprotein cholesterol; *OR,* odds ratio; *SBP,* systolic blood pressure; *TG,* triglycerides
